# Successful Treatment of Persistent Pneumothorax in a Very Preterm Neonate Using Autologous Blood Patch Pleurodesis: A Case Report From Western Odisha

**DOI:** 10.7759/cureus.88126

**Published:** 2025-07-16

**Authors:** Blesscynora Sutnga, Birendra Pradhan, F. Nasim Alli Khan, Kamal Lochan Panda

**Affiliations:** 1 Pediatrics, Veer Surendra Sai Institute of Medical Sciences and Research, Burla, IND

**Keywords:** air leaks neonate, autologous blood patch pleurodesis, persistent pneumothorax, pleurodesis, pneumothorax

## Abstract

Persistent pneumothorax is defined as pneumothorax persisting for more than five days, and it is a difficult condition to treat. Autologous blood patch pleurodesis (ABPP) is a recognized treatment technique for adults and pediatric age groups; however, limited data are available for neonates, with only one case reported from India. In our case, we report a rare and successful application of ABPP in a neonate with persistent pneumothorax lasting 14 days, marking a potentially valuable intervention pathway in similar settings.

## Introduction

While spontaneous resolution of neonatal pneumothorax is expected, what happens when it stubbornly persists and standard measures fail? That is where this case begins.

Pneumothorax is a collection of air in the pleural space, where most cases resolve spontaneously, and symptomatic cases require the insertion of an intercostal chest tube (ICT) for drainage of air [1]. However, if it persists, spontaneous resolution becomes unpredictable and often results in increased morbidity and mortality [2]. The Society of Thoracic Surgeons has defined persistent pneumothorax (PPN) as a pneumothorax that persists for more than five days [3].

There is no consensus regarding optimal management of PPN in neonates. Chemical agents, such as povidone iodine and fibrin glue, have been used for pleurodesis in neonates [4,5]. In autologous blood patch pleurodesis (ABPP), the blood of the neonate is used for pleurodesis instead of chemical agents [2]. The efficacy and safety of ABPP for PPN are well established in adults [6]. However, very limited data are available on the use of ABPP in neonates, with only a few cases described to date and one case reported from India [2]. This case report strongly favors the use of ABPP in such cases.

## Case presentation

This case involves an inborn male neonate delivered on January 10, 2025, at 30 weeks and four days of gestation, with a birth weight of 1500 g and APGAR (appearance, pulse, grimace, activity, and respiration) scores of 8 and 9 at one and five minutes of life, respectively.

The baby was admitted to the neonatal intensive care unit (NICU) at 30 minutes of life due to respiratory distress with a Silverman-Anderson score of 5/10. The chest X-ray was suggestive of respiratory distress syndrome (RDS). The neonate was initially placed on bubble CPAP (continuous positive airway pressure); however, due to a high FiO_2_ requirement (>40%) and low SpO_2_ (<90%), surfactant beractant (Survanta) was administered by the LISA (less invasive surfactant administration) method at one hour of life, as per hospital protocol. Due to rising oxygen requirements (FiO₂ > 60%) and increasing Silverman-Anderson scores, invasive ventilation was initiated with synchronized intermittent mandatory ventilation (SIMV) mode at two hours of life.

The neonate developed shock on day two with the following arterial blood gas (ABG) values: pH 7.05, pCO_2_ 15.3 mmHg, and pO_2_ 105 mmHg. He also developed pneumonia on day four of life, which was managed as per protocol. The neonate was weaned to non-invasive ventilation on day five and to oxygen via nasal cannula on day eight of life. On day 11, the patient developed severe respiratory distress, and the chest X-ray was suggestive of right-sided pneumothorax, for which an ICT was inserted and attached to a water seal bag. Blood culture grew *Citrobacter*, which was resistant to almost all common antibiotics, prompting an escalation to meropenem as per sensitivity results. With resolution of bubbling, an attempt was made to remove the chest drain on day 15 of life, but there was a recurrence of pneumothorax necessitating reinsertion of the ICT.

**Figure 1:**
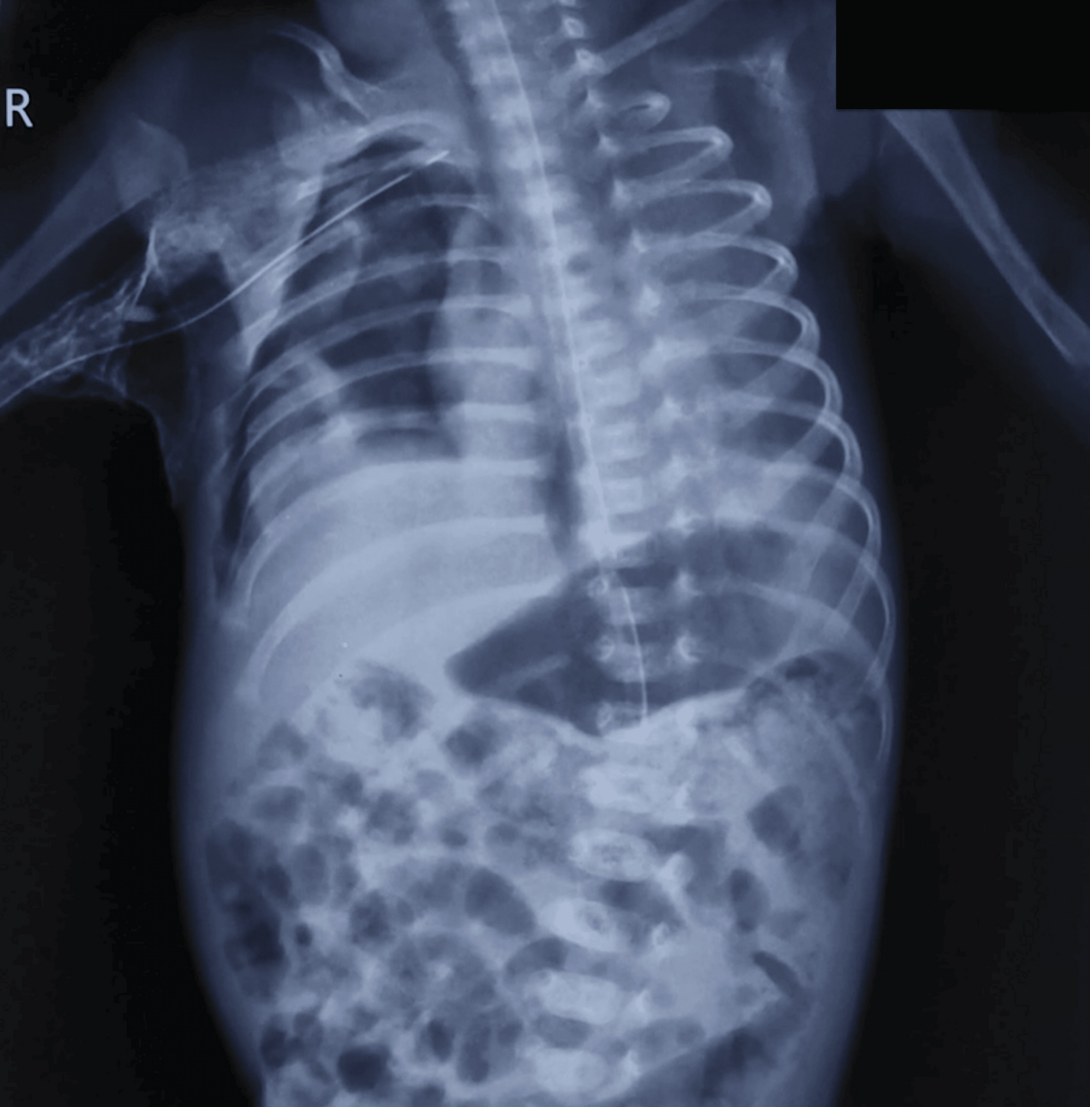
Chest X-ray on day 25 showing a persistent right-sided pneumothorax, evidenced by increased radiolucency in the right hemithorax with visible lung edge and partially collapsed right lung. Note the absence of vascular markings laterally, indicating the presence of free air in the pleural space.

The CECT (contrast-enhanced computed tomography) of the thorax done on day 21 was not suggestive of any other abnormalities like congenital pulmonary airway malformation. Given the prolonged air leak for 14 days (Figure 1), risk of infection, and parental preference to avoid chemical agents, ABPP was selected to treat the persistent pneumothorax on day 25 of life.

**Figure 2:**
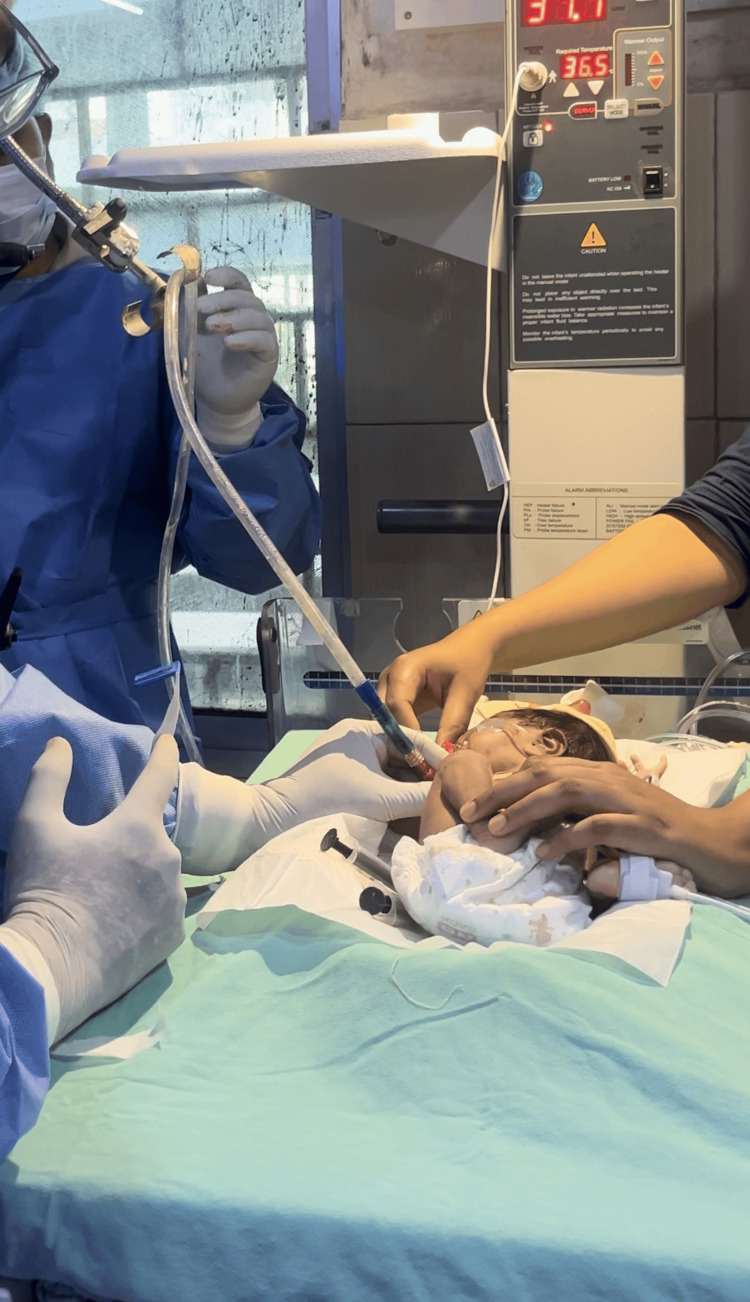
The chest tube was suspended 60 cm above the chest.

Fresh arterial blood was collected from the radial artery (3 ml/kg) and immediately injected into the pleural cavity via the chest tube, followed by injection of 5 ml of air to prevent blood from clotting in the chest tube. The chest tube was suspended 60 cm above the chest level (Figure 2) for four hours to prevent passive drainage of blood and allow pleural cavity contact, following previously published methods. The air leak was sealed within two days of ABPP, and the ICT was removed; the patient was discharged five days after ABPP.

At the last follow-up, two months after birth, the infant is experiencing adequate weight gain with no respiratory issues. While short-term follow-up was reassuring, a longer-term evaluation (e.g., pulmonary function tests, imaging) is warranted.

## Discussion

ABPP is an evidence-based, safe, and successful procedure in the adult and pediatric populations. The proposed mechanisms of ABPP include mechanical sealing of the alveolar-pleural fistula via clot formation ('blood patch effect') and the initiation of a localized inflammatory response that promotes pleural adhesion. Notably, in neonates, the inflammatory response may be less predictable due to immature immune function, which can impact the efficacy of pleurodesis [1].

During our review of the literature, we found only three case reports and one case series of ABPP reported in neonates. The first case reported from Turkey was a case of persistent pneumothorax of 17 days duration. This patient required three sessions of ABPP, with evidence of clinical improvement after 48 hours of the third patch [1]. In the second case reported from India, a persistent pneumothorax that had persisted for 10 days was treated with ABPP, and the pneumothorax resolved after 72 hours of the procedure [2]. The third case, which was reported from New Zealand, was a persistent bilateral pneumothorax of 28 days duration in an extreme preterm, which was treated with ABPP with high-frequency ventilation. The chest tube was successfully removed 72 hours later with no other complications [7]. The only case series from the USA described ABPP in three neonates, with clinical and radiological improvement in the first two cases; however, the third neonate required an extended thoracotomy and fibrin patch placement due to the persistence of an air leak [8].

In our case, ABPP was done on day 14 of persistent pneumothorax using an arterial blood patch, as described in the second case report. The use of arterial blood instead of venous blood prevents faster clotting during the procedure. We observed the closure of an air leak in a single setting of ABPP, and the pneumothorax was resolved, and the chest tube was removed after 48 hours. Unlike previously reported cases requiring multiple ABPP sessions, the single application in our patient resulted in complete resolution, possibly due to timely intervention, optimal positioning of the chest tube, and early-stage pleural responsiveness. Further study is needed to identify predictive factors for single-session success. At the two-month follow-up, the neonate exhibited age-appropriate weight gain, a normal respiratory rate, and no radiological signs of pleural thickening.

This study has some limitations. As a single case report, our findings cannot be generalized to all neonates with persistent pneumothorax. Larger studies are needed to establish standardized protocols and outcome expectations for ABPP in this population. Our follow-up duration was limited to two months, which may not be sufficient to detect long-term pulmonary complications such as pleural fibrosis or recurrent air leaks. We did not use objective lung function measurements or formal radiological scoring to assess post-procedure improvement, which may limit the precision of outcome evaluation.

## Conclusions

ABPP may be a feasible first-line option in select neonatal cases of persistent pneumothorax. This case adds to the limited but growing body of evidence that ABPP is not only feasible but also effective in neonates. Given its simplicity and low cost, it holds promise for broader use, particularly in resource-limited NICUs. There is a need for multicenter trials to standardize ABPP protocols.
